# Examination of the Effects of Conduction Slowing on the Upstroke of Optically Recorded Action Potentials

**DOI:** 10.3389/fphys.2019.01295

**Published:** 2019-10-11

**Authors:** Christopher O’Shea, Davor Pavlovic, Kashif Rajpoot, James Winter

**Affiliations:** ^1^Institute of Cardiovascular Sciences, University of Birmingham, Birmingham, United Kingdom; ^2^EPSRC Centre for Doctoral Training in Physical Sciences for Health, University of Birmingham, Birmingham, United Kingdom; ^3^School of Computer Science, University of Birmingham, Birmingham, United Kingdom

**Keywords:** optical mapping, conduction velocity, action potential upstroke, anisotropic conduction, ventricular, electrophysiology – basic

## Abstract

**Introduction:**

The upstroke of optical action potentials (APs) recorded from intact hearts are generally recognized to be slower than those recorded with microelectrodes. This is thought to reflect spatial signal averaging within the volume of tissue that makes up the optical signal. However, to date, there has been no direct experimental study on the relationship between conduction velocity (CV) and optical AP upstroke morphology in the intact heart. Notably, it is known that sodium channel block and gap junction inhibition, which both slow CV, exert differential effects on the upstroke velocity of microelectrode-recorded APs. Whether such differences are evident in optical APs is not known. The present study sought to determine the relationship between tissue CV and optical AP upstroke velocity in intact mouse hearts.

**Materials and Methods:**

Isolated, perfused mouse hearts were stained with the potentiometric dye Rh-237. Fluorescent signals were recorded from across the anterior surface of the left and right ventricles during constant pacing. Maximum rate of change in fluorescence (dF/dt_max_) and tissue CV were assessed in control conditions, during an acute period of low-flow ischemia, and following perfusion of flecainide (1–3 μmol/L), a sodium channel blocker, or carbenoxolone (10–50 μmol/L), a gap junction inhibitor.

**Results:**

During epicardial pacing, an anisotropic pattern was observed in both activation and dF/dt_max_ maps, with more rapid optical AP upstroke velocities orientated along the fastest conduction paths (and vice versa). Low-flow ischemia resulted in a time-dependent slowing of ventricular CV, which was accompanied by a concomitant reduction in optical AP upstroke velocity. All values returned to baseline on tissue reperfusion. Both flecainide and carbenoxolone were associated with a concentration-dependent reduction in CV and decrease in optical AP upstroke velocity, despite distinct mechanisms of action. Similar responses to carbenoxolone were observed for low- (156 μm pixel with) and high- (20 μm pixel width) magnification recordings. Comparison of data from all interventions revealed a linear relationship between CV and upstroke dF/dt.

**Conclusion:**

In intact mouse hearts, slowing of optical AP upstroke velocity is directly proportional to the change in CV associated with low-flow ischemia, sodium channel block, and gap junction inhibition.

## Introduction

Cardiac optical mapping, using potentiometric dyes and fluorescent-light-sensitive digital cameras, allows researchers to study the electrophysiological properties of the heart at unparalleled spatial resolution. In intact heart tissue, the morphology of action potentials (APs) recorded with optical mapping typically exhibit reduced upstroke velocity and longer rise times compared to those recorded from the same tissues with microelectrode techniques (Morad and Salama, 1979; Girouard et al., 1996; Hyatt et al., 2003; Salama et al., 2005; Bishop et al., 2007) (though not all studies agree) (Windisch et al., 1985). Meanwhile, in isolated cardiac myocytes, rates of change for optical AP upstrokes have been reported to be similar to those recorded through microelectrodes (Warren et al., 2010). The slower optical AP upstroke in intact cardiac tissue is thought to reflect the photon scattering effects of the tissue (Bishop et al., 2007, 2009), as well as the rate of conduction of electrical waves within the myocardium (Morad and Salama, 1979; Girouard et al., 1996; Hyatt et al., 2003; Salama et al., 2005; Bishop et al., 2007). Optical signals are integrated from a volume of tissue in which there is asynchronous activation and where spatial averaging of signals across and through the tissue is thought to slur the AP upstroke (Morad and Salama, 1979; Girouard et al., 1996; Hyatt et al., 2003; Salama et al., 2005; Bishop et al., 2007). Indeed, computational modeling studies suggest that the rise time of optical AP in simulated human ventricle is a non-linear function of tissue conduction velocity (CV); where slower CV equates to an increase in the time for tissue activation and therefore a slower AP upstroke (Hyatt et al., 2003). There has, however, been no experimental examination of the impact of conduction slowing on optical AP upstroke morphology in intact hearts.

Slowing of CV can occur through multiple mechanisms, including acute ischemia, tissue remodeling (hypertrophy and fibrosis), reduced sodium channel availability, and reduced gap junction coupling. Sodium channel block reduces the number of available sodium channels and decreases the transmembrane sodium current, which slows tissue CV. It is well established that concurrently with slowing CV, sodium channel block reduces the rate of maximum AP depolarization (dV/dt_max_) (Borchard and Boisten, 1982; Kojima et al., 1989; Ferrero et al., 2007). Meanwhile, gap junction inhibition slows tissue CV due to the reduction in current (source) flowing into neighboring myocytes (sink) (de Groot et al., 2003). Model studies suggest that gap junction uncoupling slows CV, yet results in increased rate of AP depolarization contrasting with observations in the presence of sodium channel blockers (Spach et al., 1987, 1988). These predictions however, are inconsistently supported (Thomas et al., 2003; Poelzing and Rosenbaum, 2004; Dhillon et al., 2013) or disputed (Jalife et al., 1989; Ozaki et al., 1989; Rohr et al., 1998; de Groot et al., 2003; Entz et al., 2016) by experimental data using a variety of experimental models, techniques, and gap junction uncoupling interventions.

In principle, the rate of change of an optical AP upstroke is a function of (1) the intrinsic AP upstroke within each individual myocyte, (2) the activation delay across and through the tissue integrated by each camera pixel, and (3) the photon scattering effects of the tissue (which increases the volume of tissue that contributes to the signal) (Bishop et al., 2009). Whether any differential effects of ischemia, sodium channel block and gap junction inhibition would be evident in optically recorded APs is not known. The present study sought to determine the relationship between tissue CV and optical AP upstroke velocity at different fractional AP levels during acute ischemia, sodium channel block, and gap junction inhibition.

## Materials and Methods

### Animal Welfare

All procedures were undertaken in accordance with ethical guidelines set out by the United Kingdom Animals (Scientific Procedures) Act 1986 and Directive 2010/63/EU of the European Parliament on the protection of animals used for scientific purposes. Studies conformed to the Guide for the Care and Use of Laboratory Animals published by the U.S. National Institutes of Health under assurance number A5634-01. Studies were approved by the University of Birmingham Welfare and Ethical Review Board.

### Optical Mapping

#### Mouse Hearts

Male mouse (C57/BL6, 25–30 g, Charles River, United Kingdom) hearts were isolated under isoflurane induced anesthesia (4% in 100% O_2_) with concomitant intraperitoneal injection of heparin (100 units injected 5-min before heart isolation). Hearts were retrogradely perfused via the aorta at a perfusion pressure of 70–80 mmHg with an oxygenated crystalloid buffer, containing (in mM); NaCl 114, KCl 4, CaCl 1.4, NaHCO_3_ 24, NaH_2_PO_4_ 1.1, glucose 11.0 and sodium pyruvate 1.0 (pH 7.4, 37^o^C). Blebbistatin was added to the perfusate at a concentration of 15 μmol/L and solutions were continuously passed through a nitrocellulose filter (5 μm pore diameter). Once contraction had abated, the potentiometric dye Rh-237 was loaded by injection into the perfusion line. 100 mL of stock solution (1.25 mg/mL in DMSO) was injected over a period of 5-min. Final DMSO concentration was 0.001%. Hearts were illuminated at 530 ± 25 nm and emitted light >630 nm was collected via an Olympus MVX10 stereomicroscope and Evolve Delta 512 × 512 EMCCD camera. Images were taken from the anterior left and right ventricular surface. Unless stated, data was collected at an acquisition sampling rate of 1 kHz with a pixel width of 156 μm.

During recordings hearts were paced with 1 ms pulses with a 110 ms cycle length from the epicardial surface at 4× the diastolic threshold using a bipolar pacing electrode (electrode spacing ∼1 mm). Recording time was 5-s. Hearts were subjected to interventions to alter ventricular CV. Namely, these were (i) 3-min of low-flow global ischemia (25% of original flow rate) (ii) increasing concentrations of the sodium channel blocker flecainide (1–3 μmol/L, total perfusion time 30–45 min) (iii) increasing concentrations of the gap junction inhibitor carbenoxolone (10–50 μmol/L, total perfusion time 30–45 min). The pacing threshold was selected to ensure electrical capture during low-flow ischemia protocols.

### Data Processing

Data processing was performed using an updated version of our freely available electrophysiological mapping software, ElectroMap^1^ (O’Shea et al., 2019), which is based in MatLab (The MathWorks). To improve the signal-to-noise of optical APs, 40-sequential beats were aligned and averaged. A region of interest encompassing the ventricles (left anterior and right) was selected and data were processed, unless otherwise stated, using a Gaussian spatial filter [5 × 5 pixel area, standard deviation (σ) = 1]. No temporal filtering was applied.

Action potential amplitude was normalized between 0 and 1. Normalization is required because signal amplitude [and so differential fluorescent amplitude (dF/dt)] is affected by heterogeneities in dye loading and tissue illumination; which is a general limitation of single-wavelength fluorescent indicators. To quantify the AP upstroke morphology, we computed the maximum differential value of the optical AP upstroke (dF/dt_max_). As described in previous studies, the fractional level that dF/dt_max_ occurs at was defined as V_F_^∗^ (Salama et al., 1994). In some studies, we also measured dF/dt at different fractional levels between 0.1 and 0.9 (dF/dt_0__.__1_ – dF/dt_0__.__9_). Unless stated otherwise, cubic spline interpolation was applied to increase the effective sampling rate from 1 to 16 kHz. Local tissue activation times were measured as the time of the maximum upstroke velocity (i.e., time of dF/dt_max_ and V_F_^∗^) (Walton et al., 2012). CV was calculated from the isochronal maps using the polynomial multi vector method with a 5 × 5 pixel area (Bayly et al., 1998; O’Shea et al., 2019).

### Analysis and Statistics

Hearts with signs of thrombi or with an initial heart rate less than 250 beats per minute were terminated and excluded. Hearts were randomized to different treatments prior to the experiments, but the nature of the paired (within heart) experimental design meant that blinding was unfeasible. However, excluding the initial determination of the region of interest, data were processed automatically with the same analytical steps and without user input, thus limiting sources of bias and error. Data analysis and statistical analysis was performed in GraphPad Prism (v8, GraphPad Software, United States). In all data presented, the mean value of dF/dt, CV or V_F_^∗^ from the entire analyzed field of view was calculated for each individual heart. The mean data (*n* = 1 for each heart) was then used for statistical tests, i.e., *n* = 14 hearts for ischemia-reperfusion, *n* = 7 for flecainide/carbenoxolone treatment, respectively. Comparisons were paired (within experiment) and thus analysis was performed using paired *t*-tests, or repeated measures one- or two-way ANOVA with Bonferroni *post hoc* tests. Statistical significance was taken as *p* < 0.05. Unless otherwise stated, data is presented as Tukey’s boxplots.

## Results

### Relationship Between Optical AP Upstroke Velocity, Conduction Velocity, and V_F_^∗^

**FIGURE 1:**
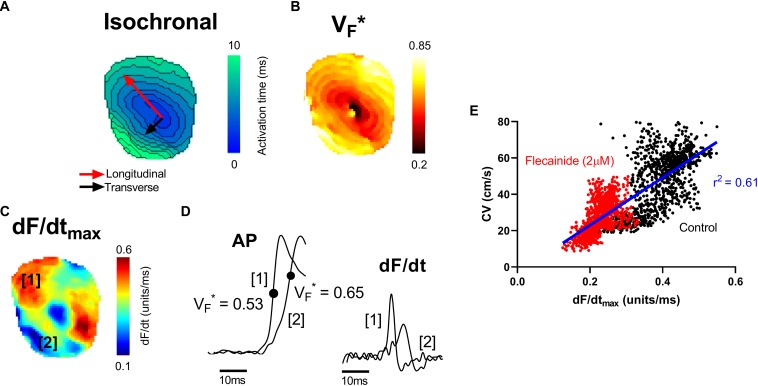
Mapping of electrical propagation in the intact heart. Representative maps from a single experiment showing different measures of electrical propagation in the intact mouse heart. **(A)** Isochronal map illustrating local tissue activation times during pacing from the central region. A pattern of anisotropic conduction can be seen, as indicated by the red and black arrows. **(B)** A similar anisotropic pattern is observed in the corresponding V_F_^∗^ map, a measure of the pattern of subsurface wavefront orientation. **(C)** dF/dt_max_ of the optical AP upstroke (signal amplitude normalized between 0 and 1). **(D)** Examples of optical AP upstroke morphology from region 1 and region 2. The corresponding derivatives are shown to the right. **(E)** Pixel by pixel correlation between dF/dt_max_ and CV before (control) and after treatment with 2 μM flecainide.

V_F_^∗^ is the fractional amplitude at which the optical AP upstroke has its fastest rate of rise and is shown in previous studies to reflect the subsurface orientation of depolarizing wavefronts as they spread through the myocardium (Hyatt et al., 2003, 2005, 2008; Zemlin et al., 2008). Here we examine the relationship between V_F_^∗^, the maximum rate of change of the optical AP (dF/dt_max_) and the pattern of electrical propagation in the intact heart. Figure 1 shows data from a representative mouse ventricle during epicardial pacing (point stimulation). Pacing was associated with an anisotropic pattern of conduction with fast (longitudinal) and slow (transverse) conduction paths. A typical oval isochronal map is shown in Figure 1A, where the faster conduction is indicated by the red arrow and the slowest conduction path by the black arrow. To the right is the corresponding V_F_^∗^ map in the same heart (Figure 1B). Consistent with previous reports, V_F_^∗^ was low (0.5 or less) along the longitudinal axis, and high (greater than 0.5) in the transverse direction (Hyatt et al., 2003, 2005, 2008; Zemlin et al., 2008).

Figure 1C shows the corresponding maximum rate of change of fluorescence (dF/dt_max_) map. dF/dt_max_ was measured as the maximum of the first derivative of the normalized optical AP trace (signal amplitude normalized from 0 to 1). Faster rates of changes are observed to align along the faster, longitudinal, conduction path. Differences in the morphology of APs in longitudinal and transverse conduction paths are shown in the example traces in Figure 1D. In keeping with previous reports, the foot of the AP was shallow along the transverse conduction path, where V_F_^∗^ is high, and steep along the longitudinal conduction path, where V_F_^∗^ is low (Hyatt et al., 2005). The corresponding dF/dt traces derived from these example APs are shown to the right. Along the faster, longitudinal, path (region 1), a larger maximum dF/dt value is present. Figure 1E shows the pixel to pixel correlation of local CV with dF/dt_max_ before and after treatment with 2 μM flecainide in this representative mouse heart (see below for further results on the effect of flecainide). A positive linear correlation is observed, with faster local CV areas exhibiting larger dF/dt_max_.

### Impact of Conduction Slowing on Optical AP Upstroke Velocity

The experiments described below were designed to quantify the effects of interventions that cause conduction slowing on the maximum rate of change of the optical AP upstroke (dF/dt_max_) and at different fractional AP amplitudes (dF/dt_0__.__1_ – dF/dt_0__.__9_).

#### Low-Flow Ischemia

**FIGURE 2:**
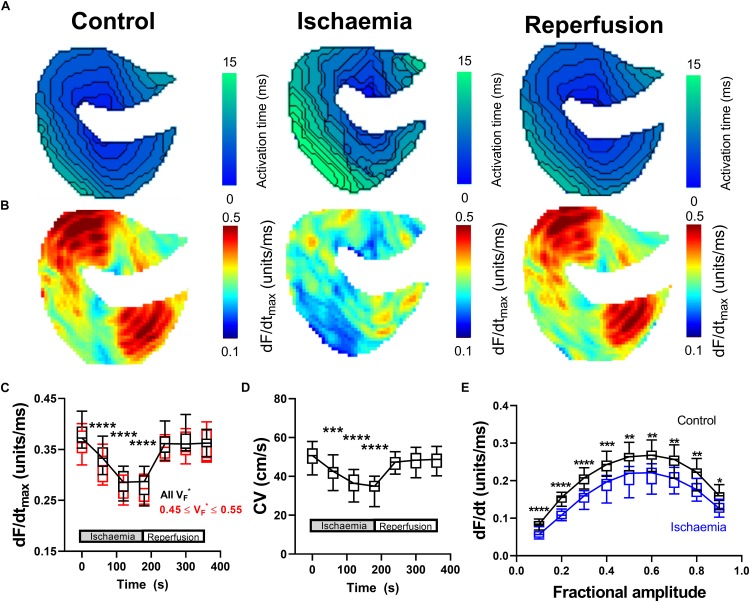
Changes in optical AP upstroke morphology during low-flow ischemia. **(A)** Representative activation maps in control conditions, during ischemia, and with tissue reperfusion. **(B)** Representative optical AP upstroke velocity maps in control conditions, during ischemia and with tissue reperfusion. Missing pixels due to location of pacing electrode. **(C)** Data from 14 hearts showing whole tissue mean changes in upstroke dF/dt_max_ at different fractional AP amplitudes during the ischemia-reperfusion protocol. Black shows data when dF/dt_max_ was calculated from all pixels, while red shows data when analysis was restricted to pixels with V_F_^∗^ between 0.45 and 0.55 to focus on areas with conduction parallel to the epicardial surface. **(D)** Corresponding changes in whole tissue mean CV. One-way ANOVA with Bonferroni correction. Time point vs. Control (time = 0 s); ^∗^*p* < 0.05, ^∗∗^*p* < 0.01, ^∗∗∗^*p* < 0.001, and ^∗∗∗∗^*p* < 0.0001. **(E)** Comparison of dF/dt at different fractional amplitudes in control conditions (time = 0 s) and during ischemia (time point 180 s). Two-way ANOVA with Bonferroni multiple comparison testing for each fractional amplitude dF/dt. Ischemia vs. Control; ^∗^*p* < 0.05, ^∗∗^*p* < 0.01, ^∗∗∗^*p* < 0.001, and ^∗∗∗∗^*p* < 0.0001.

Data presented in Figure 2 summarize the effects of a short period of low-flow global ischemia (25% of the initial flow-rate) and subsequent flow-restoration (reperfusion) on ventricular conduction in the perfused mouse heart. Reduced coronary perfusion results in the build-up of metabolic by-products, acidification of the cell, and the accumulation of potassium ions in the extracellular space; all of which drive increase in the resting membrane potential, reduce sodium channel availability and slow CV (Stanley, 2001; Klabunde, 2017). Representative activation maps during control conditions, during ischemia and following reperfusion are shown in Figure 2A. In the mouse heart, a short (3-min) period of low-flow ischemia was associated with a progressive slowing of ventricular CV as seen by a prolongation of activation time and the tightening of isochronal lines. Corresponding representative dF/dt_max_ maps are shown in Figure 2B. Slowing of CV was paralleled by a decrease in dF/dt_ma__*x*_, indicative of a slowing of optical AP upstroke velocity. Data summarizing the temporal response in dF/dt_max_ from all areas of the heart are shown in Figure 2C (black). It is well established fractional value at which dF/dt_max_ occurs (V_F_^∗^), is dependent on the transmural wave orientation, with V_F_^∗^ ∼ 0.5 reflecting parallel conduction to the epicardial surface (Hyatt et al., 2003, 2005, 2008; Zemlin et al., 2008). We hence repeated the analysis but restricted measuring dF/dt_max_ to pixels in which 0.45 ≤ V_F_^∗^ ≤ 0.55, Figure 2C (red). This did not significantly alter dF/dt_max_ response to ischemia-reperfusion.

The corresponding changes in mean tissue CV are shown in Figure 2D. Figure 2E shows the change in AP upstroke velocity during low flow ischemia at different fractional levels of the AP levels ranging from 0.1 to 0.9. A decrease in dF/dt due to ischemia is seen at all fractional levels of the AP, not just dF/dt_max_. On reperfusion, tissue CV and dF/dt_max_ gradually recovered to control values. In summary, ischemia was associated with a predictable and reversible slowing of CV that was paralleled by a decrease in the maximum rate of change of the optical AP upstroke, and at different fractional levels of the upstroke.

#### Sodium Channel Block and Gap Junction Inhibition

**FIGURE 3:**
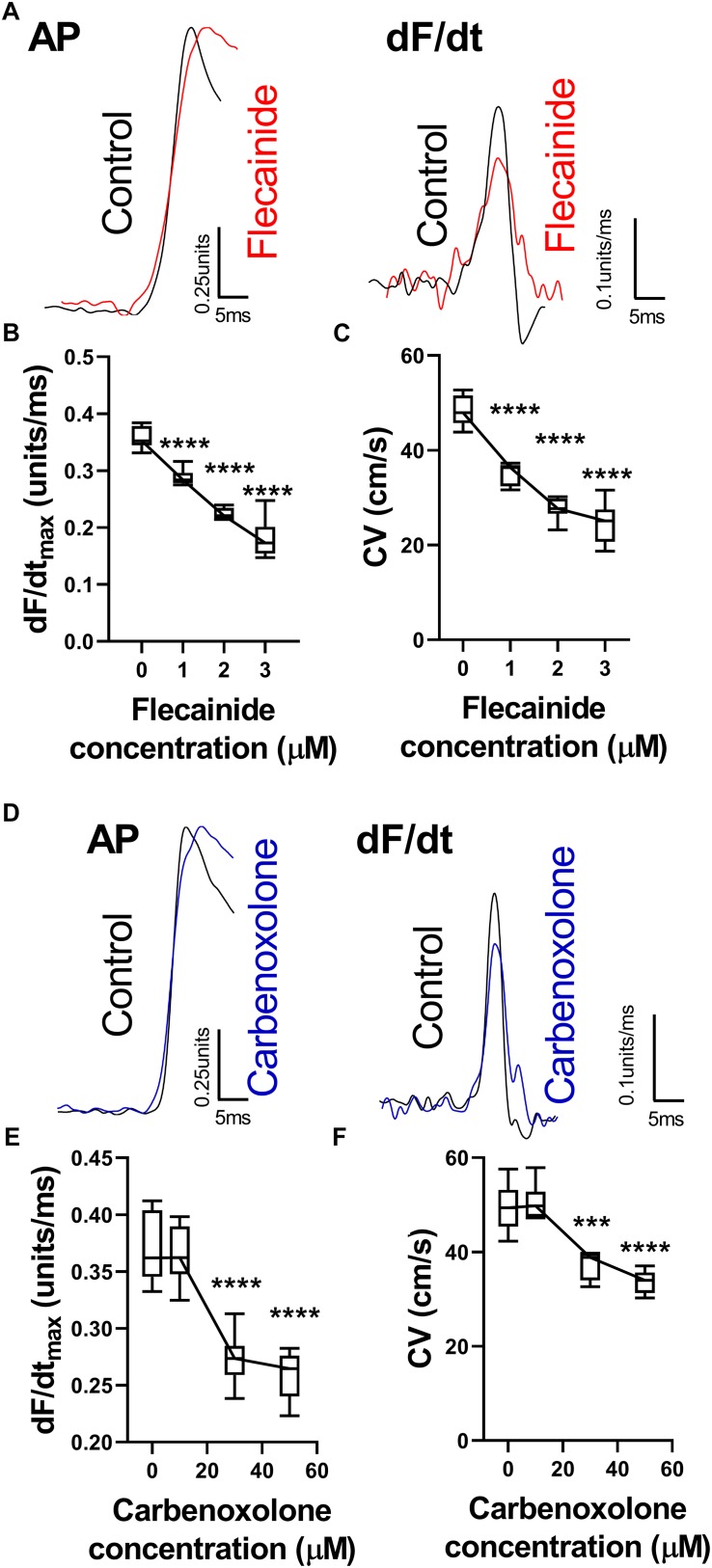
Changes in optical AP upstroke morphology with flecainide and carbenoxolone. **(A)** Representative AP upstroke morphologies and corresponding dF/dt for recordings in control conditions and with 3 μM flecainide. **(B)** Data from 7 hearts showing changes in upstroke dF/dt_max_ with increasing concentrations of flecainide. **(C)** Corresponding changes in tissue CV. One-way ANOVA with Bonferroni *post hoc* tests. Flecainide vs. Control (0 μM); ^∗∗∗^*p* < 0.001 and ^∗∗∗∗^*p* < 0.0001. **(D)** Representative AP upstroke morphologies and corresponding dF/dt for recordings in control conditions and with 50 μM carbenoxolone. **(E)** Data from 7 hearts showing changes in upstroke dF/dt_max_ with increasing concentrations of carbenoxolone. **(F)** Corresponding changes in tissue CV. One-way ANOVA with Bonferroni *post hoc* tests. Carbenoxolone vs. Control (0 μM); ^∗∗∗^*p* < 0.001 and ^∗∗∗∗^*p* < 0.0001.

The effects of reducing sodium channel and gap junction availability/conductance were examined using flecainide, a sodium channel blocker, and carbenoxolone, a gap junction inhibitor. Data are presented in Figure 3. Both flecainide and carbenoxolone were associated with a concentration-dependent slowing of ventricular CV and corresponding reduction in dF/dt_max__._ Typical AP recordings from a single pixel before and after drug perfusion are presented in Figures 3A,D for flecainide and carbenoxolone, respectively. Both drugs were associated with a decrease in amplitude-normalized dF/dt, as shown in the relative right-hand traces. Concentration-dependent slowing of dF/dt_max_ are shown in Figures 3B,E. The corresponding change in CV is shown in Figures 3C,F for flecainide and carbenoxolone, respectively.

#### Dependence of Optical AP Upstroke Velocity on Tissue CV

**FIGURE 4:**
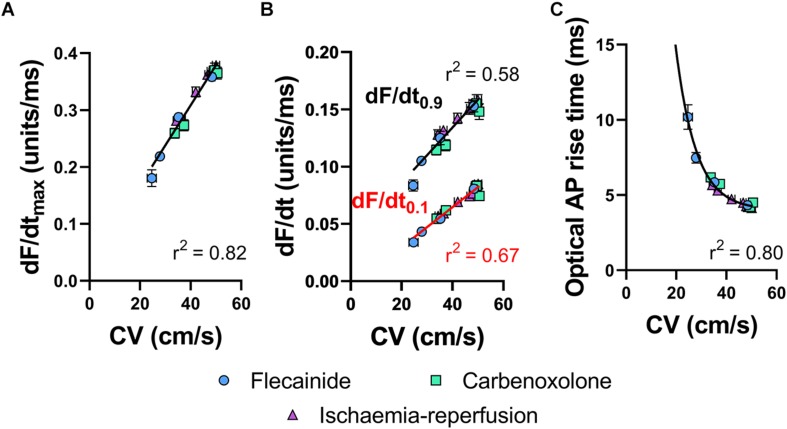
Correlations of optical AP upstroke velocity, rise time and CV. **(A)** Correlation between CV and dF/dt_max_ of the optical AP upstroke. **(B)** Correlation between CV and dF/dt_0__.__9_ (black) and dF/dt_0__.__1_ (red) and of the optical AP upstroke. **(C)** Corresponding correlation between CV and optical AP rise times (measured between 0.1 and 0.9 fractional levels of AP upstroke). **(A)** Linear fit. **(B)** Exponential. Data are mean ± SEM. Flecainide *n* = 7 hearts. Carbenoxolone *n* = 7 hearts. Ischemia-reperfusion *n* = 14 hearts.

Figure 4A shows the relationship between tissue CV and maximum optical AP upstroke velocity, including data for low-flow ischemia, flecainide and carbenoxolone protocols. Figure 4A show that in spite of their differing mechanisms of action, all data points fall on a simple linear relationship, with no obvious separation in the responses to ischemia, flecainide and carbenoxolone. The same holds for dF/dt measured at the foot (dF/dt_0__.__1_, Figure 4B red) and head (dF/dt_0__.__9_, Figure 4B black) of the AP upstroke. A non-linear relationship between tissue CV and rise-time (defined as time between 10 and 90% amplitude of the depolarization) of the optical AP was found, as shown in Figure 4C, where a non-linear exponential decay better matched the observed relationship.

### Influence of Sampling Rate and Spatial Resolution

**FIGURE 5:**
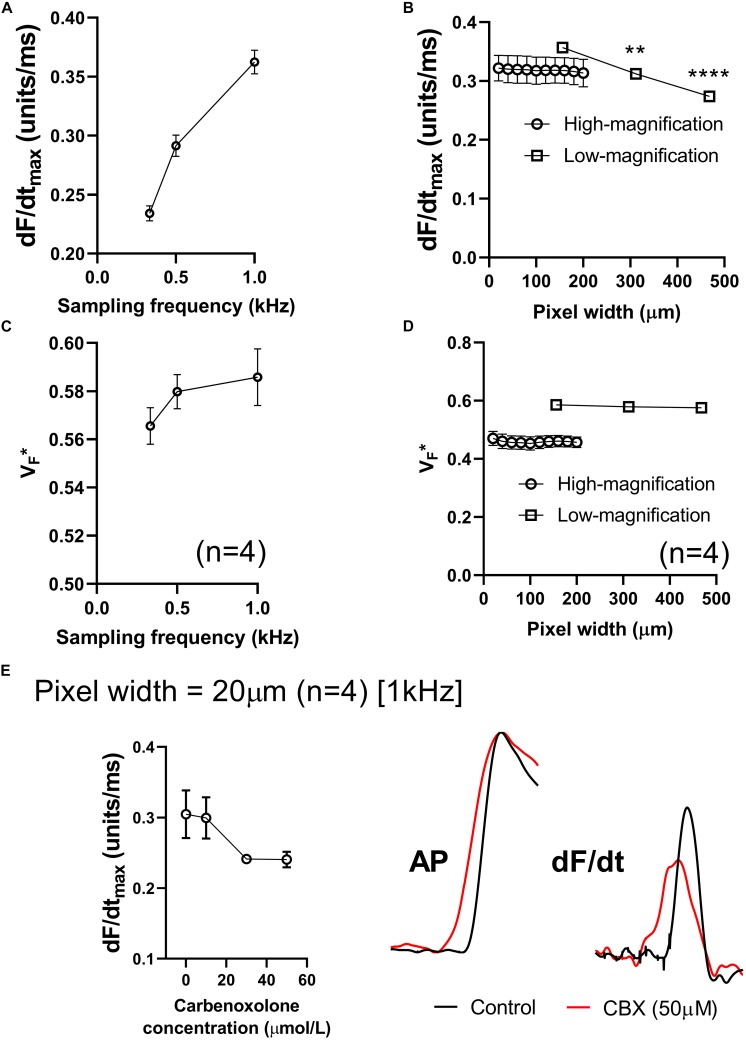
Effects of sampling frequency, magnification and pixel binning. **(A)** Effects of altering acquisition sampling frequency on maximum optical AP upstroke velocity. **(B)** Effects of pixel-binning on AP upstroke velocity at low- and high-magnification (initial pixel widths of 156 and 20 μm pixel width, respectively). **(C)** Effects of altering acquisition sampling frequency on V${}_{\text{F}}^{*}$. **(D)** Effects of pixel-binning on AP upstroke velocity at low- and high-magnification on V${}_{\text{F}}^{*}$. **(E)** Responses to carbenoxolone at high-magnification (20 μm pixel width). Data from 4 hearts with representative AP and dF/dt traces for control conditions and following carbenoxolone treatment (50 μM). Other than post processing binning (b,d), all data presented here was processed with spatial filtering and interpolation (to 16 kHz) as set out in the methods. One-way ANOVA with Bonferroni *post hoc* tests. ^∗∗^*p* < 0.01 and ^∗∗∗∗^*p* < 0.0001.

Figures 5A,B summarizes the effects of altering sampling frequency and spatial resolution (pixel width) on optical AP morphology in isolated perfused mouse hearts. Data presented in Figure 5A show a strong dependence on sampling rate, with a direct correlation between increases in acquisition sampling frequency and increased maximum optical AP upstroke velocity. Figure 5B shows the impact of post-acquisition pixel-binning on AP upstroke velocity in recordings made at low and high-magnification, equating to initial pixel widths of 156 and 20 μm, respectively. Binning pixels (2 × 2, 3 × 3…) led to a reduction in maximum optical AP upstroke velocity in recordings made with low-magnification (open circles), but not those recorded at higher-magnification (open squares) in the pixel width ranges tested. Figures 5C,D similarly summaries the results of altering acquisition sampling frequency and spatial resolution on the fractional level at which the optical AP exhibits dF/dt_max_ (V_F_^∗^). Figure 5C shows that sampling rate also effects V_F_^∗^, with increased sampling rates resulting in increased measured V_F_^∗^. Figure 5D shows that acquisition pixel width also alters measured V_F_^∗^, with lower magnification (larger pixel with of 156 μm) increasing V_F_^∗^. Pixel binning however, did not alter V_F_^∗^.

We hypothesized that at smaller pixel sizes (higher-magnification), the intrinsic AP upstroke within a single myocyte would play a more prominent role in determining AP upstroke morphology. If gap junction uncoupling by carbenoxolone does indeed increase dV/dt_max,_ we would therefore expect at higher magnification a carbenoxolone induced increase in dF/dt_max_. However, data presented in Figure 5E show this not to be the case, as carbenoxolone was associated with a concentration-dependent decrease in optical AP upstroke velocity when recording at high-magnification from the ventricular free wall (20 μm pixel width) (Figure 5E). This is opposite to observed effects of gap junction inhibition on AP upstroke in microelectrode recordings but is in keeping with data presented in Figure 3 for lower-magnification optical mapping recordings (156 μm pixel width).

### Influence of Image and Signal Processing

**FIGURE 6:**
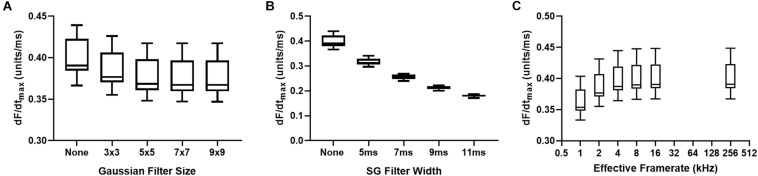
Effects of data processing on maximum optical upstroke velocity (dF/dt_max_). **(A)** Effect of gaussian spatial filter pixel size. **(B)** Effect of changing length/frame size of third order temporal Savitzky-Golay (SG) filter. **(C)** Effect of cubic spline interpolation to higher effective signal framerates. *n* = 14 hearts.

Figures 6A,B demonstrates the effects of spatial and temporal filtering on optical AP upstroke velocity. Figure 6A shows that application spatial filtering reduces dF/dt_max_. However, at kernel sizes of 5 × 5 pixels and larger (for the gaussian spatial filter applied herein), spatial filtering kernel size does not impact on measured dF/dt_max_. Temporal filtering reduces dF/dt_max_ in a frame size dependent manner. Figure 6C shows the effects of cubic spline interpolation at increasing effective sampling rates (from 1kHz acquisition sampling rate). dF/dt_max_ increases with interpolation, however, interpolation to extreme high effective sampling rates (to 256 kHz) does not substantially alter measured dF/dt_max_ values from “moderate” interpolation up to 16 kHz.

## Discussion

It has been recognized for some time that the heterogeneity of activation of the myocardium is an important determinant of the rate of change and rise time of optically recorded APs (Morad and Salama, 1979; Girouard et al., 1996; Hyatt et al., 2003; Salama et al., 2005; Bishop et al., 2007) The present study is the first to compare the effects of interventions that slow cardiac conduction on the morphology of optical APs recorded from intact hearts. Our results show that optical AP upstroke velocity is sensitive to changes in local conduction due to low-flow ischemia, sodium channel blockade and gap junction inhibition. For all study interventions, the change in maximum AP upstroke velocity was found to be directly proportional to the corresponding change in tissue CV. This finding suggests that divergent mechanism-dependent effects, shown in previous studies using microelectrode recording techniques (Borchard and Boisten, 1982; Kojima et al., 1989; de Groot et al., 2003; Ferrero et al., 2007; Dhillon et al., 2013), do not alter effects of CV changes on optically recorded AP upstroke.

During ventricular pacing, we observed a clear anisotropic pattern in optical dF/dt_max_ maps, with more rapid AP upstroke velocities aligned along the direction of the most rapid rates of conduction (and *vice versa*). This observation differs from previous reports utilizing microelectrode recording techniques, where it is established that the rate of change of the AP upstroke is slowest along the fastest conduction path (Spach et al., 1987, 1988). The biophysical basis for this observation relates to differences in intracellular coupling along the longitudinal and transverse conduction paths. With greater coupling, as occurs in the longitudinal direction, more depolarizing current flows into neighboring cells (reduced source-sink ratio) (Spach et al., 1987, 1988). The net effect is faster CV but slower cellular AP upstroke velocity. The opposite is true in the transverse direction. This phenomenon was clearly explained in seminal studies by Spach et al. (1987, 1988). However, the results of our study demonstrate that such a relationship does not hold for optical mapping recordings, where it appears AP upstroke velocity is a linear function of local CV.

It is already known that whilst optical AP upstroke velocities in single cells are comparable to those recorded with microelectrodes, in intact tissue the upstroke of the optical AP is 2–5× slower (Girouard et al., 1996; Warren et al., 2010). Slowing of the optical AP upstroke is thought to arise from the summing of signals from a volume of tissue in which there are asynchronous activation times (see later discussion on photon scattering) (Girouard et al., 1996; Bishop et al., 2007). In this paradigm, slower CV, leading to greater asynchrony in activation, would lead to a slower AP upstroke, in keeping with our experimental observations. Several findings from the present study support this interpretation. For instance, despite their differing mechanisms of action, CV and dF/dt_max_ values for ischemia, flecainide and carbenoxolone protocols all fall on a simple linear relationship. It has been suggested that gap junction inhibition slows CV in conjunction with a preserved or increased AP upstroke velocity, when recorded by microelectrode techniques. This has been reported in several studies and follows logically from the work of Dhillon et al., 2013; Spach et al., 1987, 1988; Thomas et al., 2003). There are however, several independent studies suggesting otherwise (Jalife et al., 1989; Ozaki et al., 1989; Rohr et al., 1998; Entz et al., 2016). In the present study, faster CV was associated with more rapid upstroke velocities in all interventions, strongly implying that local tissue CV, and not tissue excitability (i.e., the intrinsic upstroke velocity of single myocytes), is the major determinant of the upstroke velocity of optically recorded APs. Similar finding was found by Entz et al., 2016) in optically mapped intact guinea pig myocardium, with slowed CV induced by carbenoxolone resulting in prolonged optical rise times. Furthermore, this conclusion is indirectly supported by the findings of Hyatt et al. (2005) who found no correlation between microelectrode and optical AP upstroke velocities measured in the “same” region of isolated porcine right ventricle preparations. Our experimental results also corroborate computational predictions of a non-linear relationship between the rise time of the optical AP and tissue CV (Hyatt et al., 2003).

Notably, we found that the above experimental observations remained true even when recording data at higher-magnifications, with pixel widths as small as 20 μm, wherein carbenoxolone caused a comparable slowing of optical AP upstroke velocity as that observed in lower-magnification recordings. This is an interesting observation, as we originally hypothesized that the local AP upstroke (i.e., that of the individual myocytes) would become more dominant with smaller pixel widths, but we found this not to be the case. A likely explanation for this finding is the contribution of signals from within the ventricular wall, as well as the distortion of optical signals caused by fluorescent photon scattering within the tissue (Bishop et al., 2009). Illumination light, which excites membrane bound potentiometric dyes, penetrates in to cardiac tissue, and emitted fluorescent photons arising several hundred microns into the tissue contribute to the optical AP signal. Thus, the reduction in optical AP upstroke velocity with carbenoxolone, even when recording at high-magnification, may simply reflect the spatial differences in activation through the ventricular wall. Moreover, fluorescent photons undergo scattering events as they traverse to the tissue boundary, and due to these scattering events the photons detected on a single camera pixel originate from a widely distributed 3D tissue volume (Bishop et al., 2009). Computational models suggest that only a small proportion of the optical AP signal originates in tissues that are located geometrically beneath the recording pixel. Scattered photons contain information on the transmembrane potential at their site of origin, and so the recorded signal is the weighted average of the transmembrane potential levels within the scattered volume of tissue (Bishop et al., 2009). Our experimental data suggest that photon scattering effects dominate any local changes in AP upstroke in optical signals recorded from the mouse ventricle, even at high-magnification, and that this underpins the strong linear correlation between AP upstroke velocity and tissue CV. However, it is important again here to note the conflicting reports on the effects of gap junction uncoupling on the cellular AP upstroke, which may also explain our high-magnification findings (Jalife et al., 1989; Ozaki et al., 1989; Rohr et al., 1998; Entz et al., 2016). The mechanisms of camera tissue integration and photon scattering also explains why maximum dF/dt was correlated with regions of fastest CV, which diverges from the negative correlation observed for microelectrode recorded APs (Spach et al., 1987, 1988).

Ding et al. (2010) have previously reported on the use of maximum dF/dt of the optical AP upstroke for quantification of differences in depolarization between normal and infarct border-zone in the heart of rats post myocardial infarction. Here the authors analyzed AP upstroke without controlling for differences in optical AP amplitude, which has its limitations. Optical AP amplitude depends not only on the absolute AP amplitude in cells under the recording site, but also heterogeneities in dye loading, regional illumination, and active tissue volume. Thus, it is unclear whether the reported differences in the study of Ding et al. (2010) reflect differences in local “excitability” or simply differences in dye loading/viable tissue within the border zone. On the contrary, amplitude-normalized signals may underestimate the magnitude of conduction slowing, in scenarios where a real change in AP amplitude plays a causal role in the slowing of CV (e.g., acute myocardial ischemia). Several studies have also reported on the related measure of AP rise time (Choi and Salama, 2000; Myles et al., 2010) though not in the context of quantifying electrical conduction in the heart, but rather as a comparison of AP kinetics with traditional microelectrode recordings. Computational modeling studies have predicted that optical AP rise time is a non-linear function of CV, which was confirmed in the present experiments (Hyatt et al., 2003). In studies of anisotropic conduction, Fast and Kléber reported no difference in dV/dt (more accurately rescaled dF/dt) in optical AP recordings associated with longitudinal (slower) and transverse (faster) conduction in cultured neonatal myocyte monolayers (Fast and Kleber, 1994). This differs from the present studies observation of a correlation between anisotropic conduction patterns and dF/dt. The divergence in our results is not easily explained but may simply reflect the experimental model used (monolayers vs. intact hearts).

There has been substantive work on the contribution of subsurface signals to the morphology of the optical AP recorded in intact tissues. It is known that the fractional amplitude at which the optical AP upstroke has its maximal derivative (V_F_^∗^) is a function of the subsurface orientation of electrical wavefronts in the heart (Hyatt et al., 2003, 2005, 2008; Zemlin et al., 2008). During epicardial pacing, this largely reflects the rotation of fibers in the ventricle, which is a major determinant of anisotropic conduction (Valderrabano, 2007). The results of the present study confirm these observations in the mouse heart, reproducing the typical V_F_^∗^ patterns expected for epicardial point stimulation (Hyatt et al., 2005; Zemlin et al., 2008).

### Study Limitations

We show that AP upstroke velocity is dependent on the temporal sampling rate and spatial resolution, and for the same reason will be affected by signal processing techniques like spatial and temporal filtering and ensemble averaging. For example, we found that temporal filtering with a third order Savitizy-Goaly filter reduced baseline dF/dt_max_ values in a frame size dependent manner, and hence was not utilized. Thus, absolute AP upstroke is dependent on the experimental settings and data processing steps used. These considerations extend to use of interpolation to increase the effective sampling rate. Interpolation was applied in this study as the short timescale of the AP upstroke means dF/dt_max_ and V_F_^∗^ is likely to occur between sampling points at 1 kHz sampling frequency (1 ms frame rate). 16 kHz spline interpolation was applied as it was observed that lower effective frame rate reduced measured dF/dt_max_ values, while higher order interpolation (up to 256 kHz) did not change dF/dt_max_. However, the Nyquist theorem suggests that 1 kHz sampling rate is only sufficient to accurately resolve signal frequency content less that 500 Hz, meaning interpolation to 16 kHz may be inaccurate. Furthermore, only one interpolation method (cubic spline) was tested. Hence, further investigation is required, potentially utilizing “ground truth” computer models of optical APs in intact tissue, to optimize data processing for measurement of maximum and fractional dF/dt.

The present studies were performed in isolated mouse hearts, and additional studies are required to establish the properties of the optical AP upstroke in other species. It seems highly probable that our findings in the mouse ventricle will be more broadly applicable, though the absolute relationship between optical AP upstroke dF/dt_max_ and tissue CV will be species- and setup- dependent.

## Conclusion

In intact mouse hearts, slowing of optical AP upstroke velocity is directly proportional to the change in CV associated with low-flow ischemia, sodium channel block, and gap junction inhibition.

## Data Availability Statement

The datasets generated for this study are available on request to the corresponding author.

## Ethics Statement

The animal study was reviewed and approved by the University of Birmingham Animal Welfare and Ethical Review Board.

## Author Contributions

JW conducted the majority of studies, developed the analysis software, and conducted the analysis. CO’S integrated the software into ElectroMap and conducted the analysis. KR and DP supervised CO’S. All authors wrote and reviewed the manuscript.

## Conflict of Interest

The authors declare that the research was conducted in the absence of any commercial or financial relationships that could be construed as a potential conflict of interest.

## Footnotes

1 https://github.com/CXO531/ElectroMap

## Abbreviations

AP: action potential
CV: conduction velocity
dF/dt: rate of change in fluorescence.
